# Evaluation and analysis of respiratory infectious disease prevention behaviors in older adults

**DOI:** 10.3389/fpubh.2024.1448984

**Published:** 2024-12-11

**Authors:** Liliang Yu, Min Liu, Qing Tan, Dan Wang, Xiaoyun Chen, Mingming Zhao, Jiang Long, Mingyue Fan, Daikun Zheng

**Affiliations:** ^1^Department of Public Health and Management, Chongqing Three Gorges Medical College, Chongqing, China; ^2^Infectious Disease Control Center, Chongqing Center for Disease Control and Prevention, Chongqing, China

**Keywords:** respiratory infectious disease, old people, health literacy, KAP, disease prevention

## Abstract

**Background:**

Respiratory infectious diseases (RIDs) are a global public health problem, characterized by strong infectivity, high transmissibility, and a high incidence in the population. This study aimed to explore RID prevention behaviors among older adults and analyze their influencing factors.

**Methods:**

A cross-sectional study was conducted to examine RID prevention behaviors among 2219 Chinese older adults. Analysis of variance and the Kruskal–Wallis test were used to compare behaviors among subjects with different characteristics. Pearson's correlation analysis was used to assess the relationships between knowledge, skills, and behavior, and a generalized linear model was used for multi-factor analysis.

**Results:**

The participants in this questionnaire survey were predominantly older adults individuals aged 65–70 years (45.65%), with no more than a primary school educational level (86.70%). Univariate analysis revealed statistically significant associations between age, education, place of residence, living condition, marital status, annual income in the past year, type of medical insurance, health status, smoking status, drinking status, weekly exercise durations, and sleep status in older adults and their RID prevention behavior. Pearson's correlation analysis indicated a moderate correlation between knowledge, skills, and behavioral scores. Multivariate analysis identified place of residence, annual income, smoking habits, alcohol consumption, exercise frequency, knowledge level, and skill level as influential factors for behaviors related to the prevention and control of RID in older adults.

**Conclusion:**

Our results confirm the importance of a healthy lifestyle in RID prevention among older adults, especially in terms of smoking, alcohol consumption, and regular exercise, and provide empirical evidence for the development of health promotion programs for older adults people, particularly in rural areas.

## Introduction

Respiratory infectious disease (RID) is a group of infectious diseases caused by the invasion of pathogens from the human nasal cavity, throat, trachea, and bronchus (1). Common RIDs include influenza, tuberculosis, mycoplasma pneumonia, and the novel coronavirus pneumonia, among others. It is widely recognized that these RIDs are highly contagious and transmissible, leading to a high incidence rate within populations. Consequently, they represent a major global public health concern (2, 3).

Governments worldwide attach great importance to the prevention and control of infectious diseases, including RIDs, which are classified as national statutory infectious diseases that should be regularly reported (4, 5). In 2004, China established a national notifiable infectious diseases monitoring system, requiring medical and health institutions at all levels to report infectious diseases to the national notifiable infectious diseases reporting system in real-time (6). In 2018, more than 1.95 million cases of RID were reported in China, accounting for 25.18% of all infectious disease cases, with tuberculosis and influenza being the largest contributors (7). In the United States, more than 50 million deaths due to influenza and respiratory syncytial viruses were reported, 73.4% of which occurred among adults aged ≥ 65 years, indicating that both respiratory viruses are associated with substantial mortality in older adults (8). Furthermore, in 2013, there were more than two billion cases of upper respiratory infections and diarrheal diseases worldwide (9).

The aging of the global population is considered the most important medical and social demographic problem worldwide (10). In 2020, 9% of the world's population was aged ≥65 years. In 2019, ~1 in 11 people worldwide were 65 years and older; however, by 2050, one in six people in the world will be over the age of 65 (11). China is one of the fastest-aging countries in the world. According to China's seventh national census data, the number of people aged ≥ 60 years reached 260.4 million in 2020, representing 18.7% of the total population. In the past 10 years, the proportion of people over 60 and 65 years old has increased by 5.44 and 4.6%, respectively, marking China's entry into a period of profound aging (12, 13). Therefore, RID will affect the health of an increasing number of older adults people in China and increase the nation's disease burden (14).

As older adults individuals have weaker immune systems and a high prevalence of chronic diseases, they are highly predisposed to RID, which increases the economic burden of the disease (15). Research has indicated that individuals with a high level of health literacy are more adept at preventing infectious diseases and can considerably contribute to the mitigation of risks associated with infectious disease outbreaks (16). However, there is a paucity of research on the level of knowledge of RID, especially among older adults. In this study, we undertook a cross-sectional survey among older adults in a district and county of Chongqing, aiming to investigate the current state of their RID-related behaviors and analyze their influencing factors to provide a theoretical reference for the formulation of health intervention measures for older adults.

## Materials and methods

### Study setting and duration

In this study, the stratified cluster random sampling method was used to divide 38 townships in the Wanzhou District of Chongqing into five regions according to geographical location. In addition, a township was selected from each region by simple random sampling. Finally, people aged ≥ 65 years in five townships were selected. The study was conducted between 19 April 2023 and 25 May 2023. Before beginning the study, ethical approval was obtained from Chongqing Three Gorges Medical College of Science and Technology. All subjects provided informed consent before participating in the study, and participant confidentiality was strictly maintained throughout the study duration.

### Questionnaire

The questionnaire used in this study was revised after an in-depth review of the relevant literature (17–21) and discussion by experts, including professors and chief physicians who have long been engaged in the prevention and control of infectious diseases, public health management, and health education. The questionnaire was divided into four parts: basic demographic data, knowledge, behavior, and skills. For questions related to knowledge and skills, correct answers were given 1 point, and wrong answers were given 0 points, with the highest possible scores for these sections being 16 and 19 points, respectively. For questions related to behavior, some adopted the same scoring system as that used for the knowledge domain, whereas others were assigned different scores according to different frequencies, for the highest possible score of 14 points. Factorability was assessed using the Kaiser–Meyer–Olkin index (KMO) test and Bartlett's test of sphericity. Cronbach's α of the questionnaire was 0.914, and KMO was 0.927. Bartlett's test of sphericity (*p* < 0.0001) indicated that the scale had good reliability and validity.

Before the formal investigation, the investigators conducted questionnaire-filling training for prospective participants. After the questionnaires were collected, the investigators checked the completeness, internal consistency, and rationality of the questionnaire. Before statistical analyses, we conducted strict quality control on the questionnaire to ensure its effectiveness. Data were removed if they met one of the following criteria: (1) incomplete data; (2) anomalous data (e.g., selection of all the same options, and the illogical or disorderly selection of options); and (3) response durations of < 90 s.

### Statistical analyses

Raosoft's sample size (22) was used to determine the minimum sample size with a 95% confidence interval. The response distribution was expected to be 50%. By the end of 2022, the number of people aged ≥ 65 years in China had exceeded 200 million. Therefore, the sample size (*n*) was calculated as follows: x = *x* = *Z* (^*c*/^100) ^2^*r*(100-*r*), *n* = ^*Nx*/^((*N*-1) *E*^2^ + *x*), where *N* is the population size, *r* is the fraction of the response, and *Z* (^c^/100) is the critical value of the confidence level (c). Considering an invalid questionnaire rate of 20%, we ultimately determined that the minimum sample size was 385 individuals.

SPSS 25.0 was used to analyze the data. Categorical data are presented as frequencies and percentages, and quantitative data are presented as mean values and standard deviations (for normally distributed data) or median values with interquartile ranges (for skewedly distributed data). One-way analysis of variance (ANOVA) and the non-parametric test were used to compare behaviors among different categories of participants. Pearson's correlation analysis was used for multi-factor analyses to assess the correlations between knowledge, skills, and behavior, and for the generalized linear analysis. A *p*-value of < 0.05 was considered statistically significant.

## Results

### Basic characteristics of respondents and behavioral scores

Table 1 shows the basic characteristics and RID-related behavioral scores of the participants surveyed. After a careful quality review, 2,219 questionnaires were retained for data analysis. The respondents were predominantly aged 65–70 years (45.65%), held no more than a primary school education (86.70%), resided in rural areas (83.46%), lived with partners (56.15%), were married (72.92%), had an annual income of < 5,000 yuan (70.03%), suffered from chronic diseases (54.93%), were covered by basic medical insurance (90.54%), were non-smokers (65.71%), and were alcohol consumers (62.91%). Those with poor sleep quality and those engaging in weekly exercise were in the minority.

**Table 1:** Basic characteristics and behavioral scores of respondents.

**Characteristics**	***n***	**%**	**Behavioral scores ($\bar{x}$ ±s)**	***F***	***P***
**Age**					
65–70	1,013	45.65	8.64 ± 3.12	37.372	< 0.001
71–75	678	30.55	7.63 ± 3.04		
>75	528	23.80	7.35 ± 3.27		
**Education**					
Illiteracy	857	38.62	7.09 ± 3.16	57.893	< 0.001
Primary school	1,067	48.08	8.19 ± 2.98		
Junior high school	211	9.52	10.01 ± 2.78		
High school	56	2.52	10.45 ± 2.78		
College degree or above	28	1.26	10.57 ± 3.35		
**Place of residence**					
City	367	16.54	8.82 ± 3.26	27.791	< 0.001
Rural	1,852	83.46	7.87 ± 3.14		
**Living condition**					
Live alone	421	18.97	7.34 ± 3.30	9.757	< 0.001
With partner	1,246	56.15	8.18 ± 3.11		
With children	227	10.23	7.6 ± 3.03		
With partner and children	290	13.07	8.59 ± 3.17		
Others	35	1.58	8.91 ± 3.69		
**Marital status** ^*^					
Never married	96	4.33	7.32 ± 3.44	20.558	< 0.001
Married	1,618	72.91	8.31 ± 3.13		
Divorce	383	17.26	7.51 ± 3.22		
Widowed	122	5.50	6.39 ± 2.76		
**Annual income** ^*^					
0–2,999	874	39.39	7.58 ± 3.19	45.426	< 0.001
3,000–4,999	680	30.64	7.64 ± 2.84		
5,000–7,999	329	14.83	8.04 ± 3.13		
8,000–9,999	154	6.94	9.16 ± 3.43		
>10,000	182	8.20	10.63 ± 2.76		
**Chronic patients**					
Yes	1,219	54.93	8.04 ± 3.17	0.074	0.786
No	1,000	45.07	8.01 ± 3.20		
**Medical insurance**					
Employee medical insurance	106	4.78	10.81 ± 2.66	23.942	< 0.001
Basic medical insurance	2,009	90.54	7.92 ± 3.13		
Commercial insurance	29	1.30	7.93 ± 2.88		
Self-financing	69	3.11	6.93 ± 3.32		
Others	6	0.27	7.5 ± 3.67		
**Health status**					
Very good	165	7.44	9.72 ± 2.99	23.091	< 0.001
Good	722	32.53	8.23 ± 3.05		
General	878	39.57	8.01 ± 3.19		
Poor	354	15.95	7.03 ± 3.15		
Very poor	100	4.51	7.35 ± 3.11		
**Smoking**					
No	1,458	65.71	8.35 ± 3.15	3.775	0.023
Yes	669	30.15	7.37 ± 3.14		
Quit smoking	92	4.14	7.6 ± 3.26		
**Drinking**					
No	1,396	62.92	8.31 ± 3.20	16.198	< 0.001
Yes	753	33.93	7.6 ± 3.10		
Quit drinking	70	3.15	6.97 ± 3.00		
**Weekly exercise duration** ^*^					
Never	1,232	55.51	7.6 ± 3.22	16.9	< 0.001
1–2 times	388	17.49	8.62 ± 2.72		
3–4 times	337	15.19	8.57 ± 2.81		
5 or more times	262	11.81	8.44 ± 3.76		
**Sleep quality**					
Very good	214	9.65	8.97 ± 3.15	15.519	< 0.001
Good	770	34.70	8.4 ± 3.14		
General	810	36.50	7.79 ± 3.12		
Poor	328	14.78	7.5 ± 3.21		
Very poor	97	4.37	6.68 ± 3.05		

*F, F*-statistic in ANOVA; *P* < 0.05 was considered statistically significant.

^*^Stands for non-parametric test.

ANOVA and the non-parametric test were used to analyze differences in RID prevention behaviors among older adults. Statistically significant associations between age, education, place of residence, living condition, marital status, annual income in the past year, type of medical insurance, health status, smoking status, drinking status, weekly exercise duration, and sleep status in older adults, and RID prevention behaviors were observed (*p* < 0.05).

### Correlation of knowledge, skills, and RID-related behaviors

Table 2 shows the correlation between knowledge and skills, and RID prevention behaviors among older adults. Each variable's skewness and kurtosis were < 2 and 7, respectively, indicating approximately normal distributions. Pearson's correlation analysis revealed that there were moderate correlations between knowledge and skills, and behavioral scores (*r* > 0.5, *p* < 0.05).

**Table 2:** Correlation analysis of knowledge, skills, and behavioral scores related to RID.

**Group**	** $\bar{x}$ **	***s***	**Skewness**	**Kurtosis**	**Knowledge score**	**Skill score**
Knowledge score	9.1	3.98	−0.499	−0.566	1	–
Skill score	9.06	4.84	0.307	−0.616	0.575^**^	1
Behavioral score	8.03	3.18	−0.145	−0.683	0.520^**^	0.582^**^

$\bar{x}$, sample mean; S, sample standard deviation.

^**^P < 0.05.

#### Correlates of RID-related behaviors

A generalized linear regression model was used to identify factors associated with RID-related behaviors in older adults. As shown in Table 3, those from urban backgrounds had higher behavioral scores than those from rural backgrounds, and income level was inversely proportional to behavioral scores. Current and former smokers had lower behavioral scores than those who never smoked. Similarly, current and former alcohol drinkers had lower behavioral scores than never-drinkers. The behavioral scores of those who participated in weekly exercise were higher than those who did not. Finally, knowledge and skill scores were directly proportional to behavioral scores.

**Table 3:** Correlates of RID-related behaviors in older adults based on the multilevel logistic regression model.

**Predictors**	***B***	**SE**	**95% CI**		**Hypothesis testing**	
			**L**	**U**	**Wald**	**P**
**Place of residence**						
City	0.392	0.145	0.107	0.676	7.272	0.007
Rural	–	–	–	–	–	–
**Annual income**						
0–2,999	−2.001	0.202	−2.396	−1.606	98.646	< 0.001
3,000–4,999	−1.704	0.206	−2.107	−1.301	68.71	< 0.001
5,000–7,999	−1.185	0.224	−1.624	−0.745	27.909	< 0.001
8,000–9,999	−0.739	0.261	−1.25	−0.228	8.031	0.005
>10,000	–	–	–	–	–	–
**Smoking**						
No	−0.553	0.128	−0.805	−0.302	18.567	< 0.001
Yes	−0.864	0.289	−1.431	−0.298	8.945	0.003
Quit smoking	–	–	–	–	–	–
**Drinking**						
No	−0.372	0.127	−0.621	−0.124	8.629	0.003
Yes	−0.906	0.325	−1.542	−0.27	7.8	0.005
Quit drinking	–	–	–	–	–	–
**Weekly exercise duration**						
Never	0.495	0.144	0.214	0.776	11.915	0.001
1–2 times	0.13	0.152	−0.168	0.427	0.729	0.393
3–4 times	−0.731	0.169	−1.062	−0.4	18.753	< 0.001
5 or more times	–	–	–	–	–	–
**Knowledge score**	0.23	0.017	0.198	0.263	194.289	< 0.001
**Skill score**	0.231	0.014	0.203	0.259	257.676	< 0.001

B, Regression coefficient; SE, standard error; 95% CI, 95% confidence interval; L, lower confidence limit; U, upper confidence limit.

## Discussion

Research findings indicate that early screening of older adults people with low levels of RID-related health literacy is crucial, as RID can lead to aggravated illness among older adults patients with chronic diseases. Similarly, good RID prevention behaviors are key to RID prevention in older adults (23, 24). In this study, we assessed the RID-related behaviors of 2,219 Chinese older adults people aged ≥ 65 years and identified the factors influencing these behaviors.

Based on our results, knowledge and skill scores were moderately correlated with RID-related behavioral scores among older adults. Factors associated with RID prevention and control behaviors included residence, annual income, smoking, drinking, exercise, knowledge, and skills. These results align with the findings of Sun et al., who investigated the health literacy of rural residents related to infectious diseases. They found that approximately half of the respondents had limitations, with educational level and access to health information being associated factors (25). Our study found that, consistent with the findings of Chen et al. and Zhang et al., older adults in urban areas had significantly higher levels of RID health literacy than those in rural areas, possibly because of the paucity of good health services and access to health information for rural residents (26, 27). In addition, RID knowledge level had a direct impact on RID prevention behavior, where low RID knowledge levels led to poor prevention measures, which is consistent with the findings of Wang et al. (20) and Honarvar et al. (28). Therefore, public health knowledge should be promoted to improve the knowledge level of RID among older adults in remote areas and those with low education levels and cultivate more effective prevention behaviors. In addition, studies have shown the potential impact of a healthy lifestyle on RID. Consistent with our findings, smoking and alcohol consumption significantly increased the risk of RID (29–32), whereas increased physical activity reduced the risk of RID (33).

According to the Action Plan for a Healthy Lifestyle for All People (2017–2025) issued by the Chinese government, special actions to encourage moderate exercise, smoking control, alcohol restriction, and mental health will help to spread health knowledge, and improve individuals' health awareness and behavioral ability. Interestingly, this study found that older adults individuals who did not smoke did not drink, and exercised every week had the highest RID prevention and control behavioral scores, indicating that behaviors associated with healthy living are conducive to the prevention and control of RID in rural older adults people. Therefore, medical workers should offer guidance for a healthy lifestyle, pay attention to changes in smoking and drinking behaviors, and take reasonable measures to improve RID prevention by engaging the public. Additionally, based on the health education needs of the rural older adults, teams of family doctors should implement targeted and personalized health education for this demographic. For example, for older adults individuals who are less educated, health information should be frequently disseminated in the field in an easily understood dialect. For those with poor vision and memory, health education should be carried out by means of radio or audio resources. Finally, older people who live alone or are divorced can participate in health promotion activities together.

This study revealed the influencing factors of RID knowledge and behavior among older adults, providing a basis for improving their health literacy. However, our study had some limitations that are worth mentioning. First, the questionnaires we collected underwent strict quality control to ensure the authenticity of the data as much as possible. However, the research data comprised the subjective responses of the respondents, which may be subject to information bias, especially for questions related to sleep quality and health status. In addition, the inability to effectively adjust for confounding variables in the model may have introduced bias into the results.

In summary, our study confirmed the importance of a healthy lifestyle for the prevention of respiratory infections in older people, especially in terms of smoking, alcohol consumption, and regular exercise, providing evidence for the development of health promotion programs for rural, older people.

## Data Availability

The original contributions presented in the study are included in the article/Supplementary material, further inquiries can be directed to the corresponding authors.
